# A systematic review of tagging as a method to reduce theft in retail environments

**DOI:** 10.1186/s40163-017-0068-y

**Published:** 2017-05-30

**Authors:** Aiden Sidebottom, Amy Thornton, Lisa Tompson, Jyoti Belur, Nick Tilley, Kate Bowers

**Affiliations:** UCL Department of Security and Crime Science, 35 Tavistock Square, London, WC1H 9EZ UK

## Abstract

**Background:**

Retailers routinely use security tags to reduce theft. Presently, however, there has been no attempt to systematically review the literature on security tags. Guided by the acronym EMMIE, this paper set out to (1) examine the evidence that tags are effective at reducing theft, (2) identify the key mechanisms through which tags are expected to reduce theft and the conditions that moderate tag effectiveness, and (3) summarise information relevant to the implementation and economic costs of tagging.

**Methods:**

In this mixed-methods review, we performed systematic keyword searches of the published and unpublished literature, hand searched relevant journals, conducted forward and backward citation searches and consulted with four retailers. Studies were included if they reported an explicit goal of reducing the theft or shrinkage of items through the use of security tags in retail environments.

**Results:**

We identified 50 eligible studies, eight of which reported quantitative data on the effectiveness of tags in retail environments. Across these eight studies, five showed positive results associated with the introduction of tags, but heterogeneity in the type of tag and reported outcome measures precluded a meta-analysis. We identified three mechanisms through which tags might plausibly reduce theft—increase the risks, reduce the rewards, increase the effort—which were found to vary by tag type, and their activation dependent on five broad categories of moderator: retail store and staff, customers (including shoplifters), tag type, product type, and the involvement of the police and criminal justice system. Implementation challenges documented in the literature related mainly to staffing issues and tagging strategy. Finally, although estimates are available on the costs of tagging, our searches identified no high-quality published economic evaluations of tagging.

**Conclusions:**

Through applying the EMMIE framework this review highlighted the complexity involved in security tagging in retail environments, whereby different kinds of tags are expected to reduce theft through different casual mechanisms which are dependent on a distinctive configuration of conditions. Based on the available evidence it is difficult to determine the effectiveness of tags as a theft reduction measure, albeit there is suggestive evidence that more visible tags are associated with greater reductions in theft than less visible tags.

**Electronic supplementary material:**

The online version of this article (doi:10.1186/s40163-017-0068-y) contains supplementary material, which is available to authorized users.

## Background

Shoplifting is a persistent problem for many retailers. It is a major source of ‘shrinkage’, the umbrella term used to denote preventable losses attributed to theft, fraud, error, damage or wastage (Beck 2016a). According to estimates from the *Global Retail Theft Barometer* (2015), the cost of retail crime globally exceeded US $214 billion in 2014–15.^1^ Beyond obvious financial losses to retailers, the effects of retail crime can be far reaching. In extreme cases, chronic crime levels can force businesses to close thereby limiting employment opportunities and the availability of goods and services (Hopkins and Gill 2017). Moreover, the costs of high crime levels ultimately fall on the consumer through elevated prices, comprising what Bamfield and Hollinger (1996) call a ‘crime tax’.

Loss prevention is thus a key concern for many retailers (Hayes 1997). It is also big business: global expenditure on loss prevention is estimated to be around 0.65% of total sales (Global Retail Theft Barometer 2015). Diverse measures are implemented to prevent losses in retail environments. These include “store detectives and guards, active customer service initiatives, secure product handling procedures, locked or otherwise specialized display fixtures, reinforced packaging, staff screening and training, in-store signage… periodic audit/cycle counts, cabling, sales floor design, civil and criminal sanctions, display alarms, and CCTV video domes” (Hayes and Blackwood 2006, p. 263). Despite the preponderance of security measures used by retailers, evaluations of their effectiveness remain scarce (Hopkins and Gill 2017). Those evaluations that are available have also been criticised for, amongst other things, insufficient time periods over which to assess the impact of interventions and failure to identify the causal mechanism(s) through which security devices produce their effects (Hopkins and Gill 2017).

The focus of this review is on the application of security tags in retail environments. Tags are widely used in retail settings (DiLonardo 2015; Hayes 2007; Beck and Palmer 2010; Global Retail Theft Barometer survey 2015). They are often favoured over other loss prevention methods because tagged products remain on display and are accessible to staff and prospective buyers. Despite the popularity of tagging, to date there has been no attempt to systematically review the evidence on whether they are effective at reducing theft. In this paper, informed by EMMIE—an acronym denoting five categories of evidence considered relevant to crime prevention decisions makers (Johnson et al. 2015)—we summarise the available evidence to: (1) determine whether tags are **E**ffective at reducing theft; (2) articulate the **M**echanisms through which tags are expected to reduce theft and the conditions that **M**oderate tag effectiveness; and (3) identify the **I**mplementation considerations and **E**conomics of tagging.

The remainder of this paper is organised as follows. First, we briefly chart the history and development of tagging in retail environments. Next, we describe the acronym EMMIE and how it informed this review. Third, we report our methods and search strategy. The results then follow, organised according to EMMIE. We finish by discussing our findings and their implications.

## On the design and development of security tags

‘Tags’ is a convenient umbrella term for a diverse range of security products including bottle caps, spider wraps and anti-tamper seals (see Beck 2016b). This review focuses on two specific categories of tag. The first are *ink tags*, which refer to reusable ‘hard tags’ that contain glass phials of indelible ink or dye that is expelled when the tag is tampered with, thereby rendering the product damaged and less desirable (DiLonardo and Clarke 1996). Ink tags are non-electronic. They are typically used by apparel manufacturers and tend to be removed by cashiers at point of sale. Ink tags originated in Sweden in the 1980s. Usage was initially patchy: tags were often large and bulky and application and removal was challenging (DiLonardo 2008). Progressive refinements to the design of ink tags resulted in a greater penetration rate, particularly in the USA.

A second broad category of security tags is *Electronic Article Surveillance* (EAS) tags. These can take several forms, from “hard” plastic tags to “soft” self-adhesive paper tags (DiLonardo 2008, 2015; Hayes 2007). EAS systems generally consist of three components: the electronic tag, detector gates with built-in radio antennae (typically located at store exits) and a control unit (Bamfield 1994). EAS tags sound an alarm if they pass the detector gates without being removed or de-activated. EAS tags operate on various parts of the radio wave spectrum from electro-magnetic (EM) to acousto-magnetic (AM) or radio frequency (RF), depending on the manufacturer (DiLonardo 2015).

Like ink tags, EAS tags have undergone considerable technological innovation over the past 50 years since their inception. Whilst EAS tags were originally designed for apparel retailers, in response to widespread thefts they have since been applied to a much wider range of goods, including groceries and music products. The first commercial tags deployed in the 1960s were hard, round and plastic, attached by pins, using RF, EM and microwave technologies (DiLonardo 2015). The 1980s saw the advent of smaller magnetic “soft” EAS tags which were disposable, attached with adhesive backs, and could be deactivated at point of sale. The 1990s produced tags which could be sewn into or heat-sealed onto items of clothing at the point of manufacture (DiLonardo 2015). This process of *source*-*tagging* has become increasingly popular over the past decade, particularly among retailers since it ensures better consistency in tag application and it removes the requirement of retailers to train and resource staff to tag items in store (Beck and Palmer 2010). More recently, retailers have experimented with the use of RFID EAS tags albeit primarily as a way of monitoring stock levels as opposed to controlling theft (see Jones et al. 2005). EAS tags are arguably the most commonly used contemporary article surveillance measure, boosted by ever-cheaper RF technology. Seventy-three per cent of respondents to the *Global Retail Theft Barometer* survey (2015) reported using EAS tags.^2^

## EMMIE and our approach to systematic review

In this review we used the acronym EMMIE as our guiding framework (Johnson et al. 2015). EMMIE does not mandate a preferred method of undertaking a systematic review. However, Johnson et al. (2015) do suggest that evidence that reliably speaks to the five dimensions of EMMIE might best be captured through a mixed methods design. This can be seen in the EMMIE-informed review of alley gating by Sidebottom and colleagues (2017). In their review, questions concerning the effectiveness of alley gating—what works?—were examined using meta-analytic methods, whereby quantitative data from primary evaluation studies were pooled to produce an overall effect size. By contrast, questions on *how* alley gates are expected to reduce crime (mechanisms) and under what conditions (moderators) were examined using a qualitative approach inspired by realist review methods (see Pawson 2006). This involved a wider range of primary studies, including but not limited to those evaluative studies that were eligible for meta-analyses, being read, coded and discussed with the aim of formulating working theories on the causal processes through and conditions under which alley gates may produce their observed effects. In this review, consistent with Johnson et al. (2015) and Sidebottom et al. (2017), we adopt a mixed-methods approach.

## Methods

### Criteria for considering studies for this review

We used the following criteria in selecting studies for this review:

- *The study must report an explicit goal of reducing the theft, shrinkage or loss of items through the use of security tags*. Theft could refer to offences committed by customers or employees, although in many cases we expect the offender will be unknown. ‘Tag’ can refer to any type of article surveillance measure including ink tags, electronic tags or more recent hybrid tags. Studies were included irrespective of who funded or implemented the tags (such as tag vendors, police, retailers), or whether they were implemented in isolation or as part of a wider package of loss prevention measures.
- *The study must relate specifically to retail environments, defined here as physical spaces open to the public where merchandise is sold*. This is distinguished from tags implemented in non-retail environments (such as the workplace) or the retail supply chain, both of which were excluded from this review. Studies in which tags were attached at source (by the manufacturer) or in-store (by the retailer) were included.

Consistent with other EMMIE-informed reviews (Sidebottom et al. 2017), we used a mixed-methods approach when synthesising evidence according to the five categories of EMMIE. To determine the effectiveness of tags, we selected studies that satisfied points (a) and (b) above and met the following two criteria:

c. *The study must report at least one quantitative theft, shrinkage and/or loss outcome measure*. Retailers differ in how they define and measure shrinkage (see Beck 2006, 2016a). For this reason, we accepted a range of quantitative outcome measures that relate to the effectiveness of tags including but not limited to theft based on police recorded data.
d. *The study must report original research findings.* Quantitative findings for any study were incorporated only once, even if reported in multiple publications. Where this was the case, the study reporting the most detailed information was included.

Based on an initial scan of the literature, we anticipated a small number of tag impact evaluations. Consequently, in this review we considered various research designs (including simple before and after designs). However, as will become clear, in the event all but one of the identified evaluations of tagging in retail environments used some form of comparison group.

Items (c) and (d) were not part of the inclusion criteria for selecting studies that may provide evidence concerning the **M**echanisms, **M**oderators, **I**mplementation and **E**conomics of tags. For these elements of EMMIE, we undertook a realist-inspired review and therefore considered a broader range of studies. To be included in this branch of our review, studies had to satisfy points (a) and (b) above—report an explicit goal of reducing theft, shrinkage or loss in retail environments through the use of tags—and report substantive information relating to at least *one* of the items below:

e. Theft-related causal mechanisms activated by tags in retail environments;
f. The conditions judged to influence the activation of theft-related causal mechanisms in retail environments;
g. The implementation of tags in retail environments; or
h. The costs of tags in retail environments.

Note that for this branch of our review we used generous inclusion criteria and considered studies to be eligible if they “reported” information relevant to Mechanisms, Moderators, Implementation or Economics; eligibility was not contingent on studies providing *empirical evidence* pertaining to these elements. Based on previous realist reviews in criminology (van der Knaap et al. 2008), it was felt that insisting on this more stringent threshold would be too restrictive and result in the exclusion of potentially informative studies.

### Identifying studies: databases and information sources

Eligible studies were sought using five methods: (1) A keyword search of electronic databases (see Additional file 1: Appendices 1, 2 and 3)^3^; (2) a hand search of relevant journals not included in the databases examined^4^; (3) a keyword search of publications by relevant government, research and professional agencies (see Additional file 1: Appendices 4 and 5); (4) forward and backward citation searches of evaluation studies included in “Effect” section^5^; and (5) consultation with retailers and loss prevention managers (see “Consulting retailers”). We considered the last tactic important to identify what we expected to be a substantial grey literature on the effectiveness of tags produced for specific businesses but treated as commercially sensitive. No date restrictions were applied to our searches. Studies did, however, have to be available in English. Our list of candidate studies was checked by recognised experts on retail crime (see Additional file 1: Appendix 6).

### Consulting retailers

Retailers were approached in two ways. Firstly, via the UK *Metropolitan Police Service (MPS) Business Crime Hub*, which coordinates and provides crime prevention advice to many large retailers in London. Twenty-three retailers were sent an e-letter (see Additional file 1: Appendix 7) by the MPS outlining the purpose of our review and requesting that they participate in the study, specifically through the sharing of information gleaned from any trials of tagging which they had been involved in. A copy of the review protocol was also attached to the email. Secondly, meetings were held with senior police officers who at the time of writing held the positions of national and deputy lead for retail crime in England and Wales. Both were told of this review and asked to circulate a copy of the aforementioned email to relevant retailers requesting their participation.

### Data extraction and management

For those studies eligible for inclusion, two researchers independently extracted relevant information. This information related both to the characteristics of the study (author, date, setting) and to the different elements of EMMIE (see Additional file 1: Appendix 8). Any disagreements were resolved through discussion with the research team.

### Assessment of bias in eligible studies included in our “Effect” section

In an attempt to quantify methodological probity, all studies that made causal claims about the effectiveness of tags (i.e., those included in “Effect” section) underwent evidence appraisal, conducted independently by two authors. Four potential sources of bias were assessed: (1) selection bias (whether action and control groups (where appropriate) were comparable at baseline), (2) measurement bias (the extent to which the data analysed were a reliable measure of theft as opposed to shrinkage), (3) regression to the mean (whether the installation of tags followed a sudden increase (or decrease) in theft) and (4) contamination effects (the extent to which study authors identified and/or discounted factors that might plausibly explain the outcome patterns observed). Each domain was scored as low risk, medium risk or high risk. High risk of bias is taken here to mean no mention of the above issues and/or how they might affect the reliability of the findings. Medium risk denotes mention of relevant issues but no attempt to discount them. Low risk of bias denotes mention of relevant issues and statistical efforts to discount them. Any disagreements were resolved through discussion with the research team. It is important to emphasise that our assessment of any bias that might be present in these studies may relate more to their descriptive validity (what is reported) than their internal validity (Farrington 2003). This is most relevant to our ‘high risk’ label, which was awarded not only for methodological weaknesses but also where insufficient information was provided to make a determination about methodological quality.

### Realist review

As part of our realist review, four researchers read and independently coded those articles judged relevant to tagging. A code set was created to extract information on Mechanisms, Moderators, Implementation and Economics, and was used to develop working theories as to how tags operate as a theft reduction method. These theories were then scrutinised and refined through regular group discussions. Consultation with retailers and loss prevention managers provided supplementary information and a means of receiving feedback on the emerging theories.

## Results

### Search results and screening

Our searches returned over 1000 potentially eligible records (once duplicates were removed). The title and abstract of identified studies were screened by three review authors to determine eligibility based on our inclusion criteria. Tests of inter-rater reliability were carried out to ensure the accuracy of this process with 92% agreement on inclusion and exclusion. Our approach at this stage erred on the side of inclusivity, with studies being retained if the title and abstract made any reference to tagging in retail environments. The full text of 152 studies was then examined by the same three review authors using our inclusion criteria. Disagreements were resolved by discussion and, where necessary, through the involvement of additional authors.

**Fig. 1:**
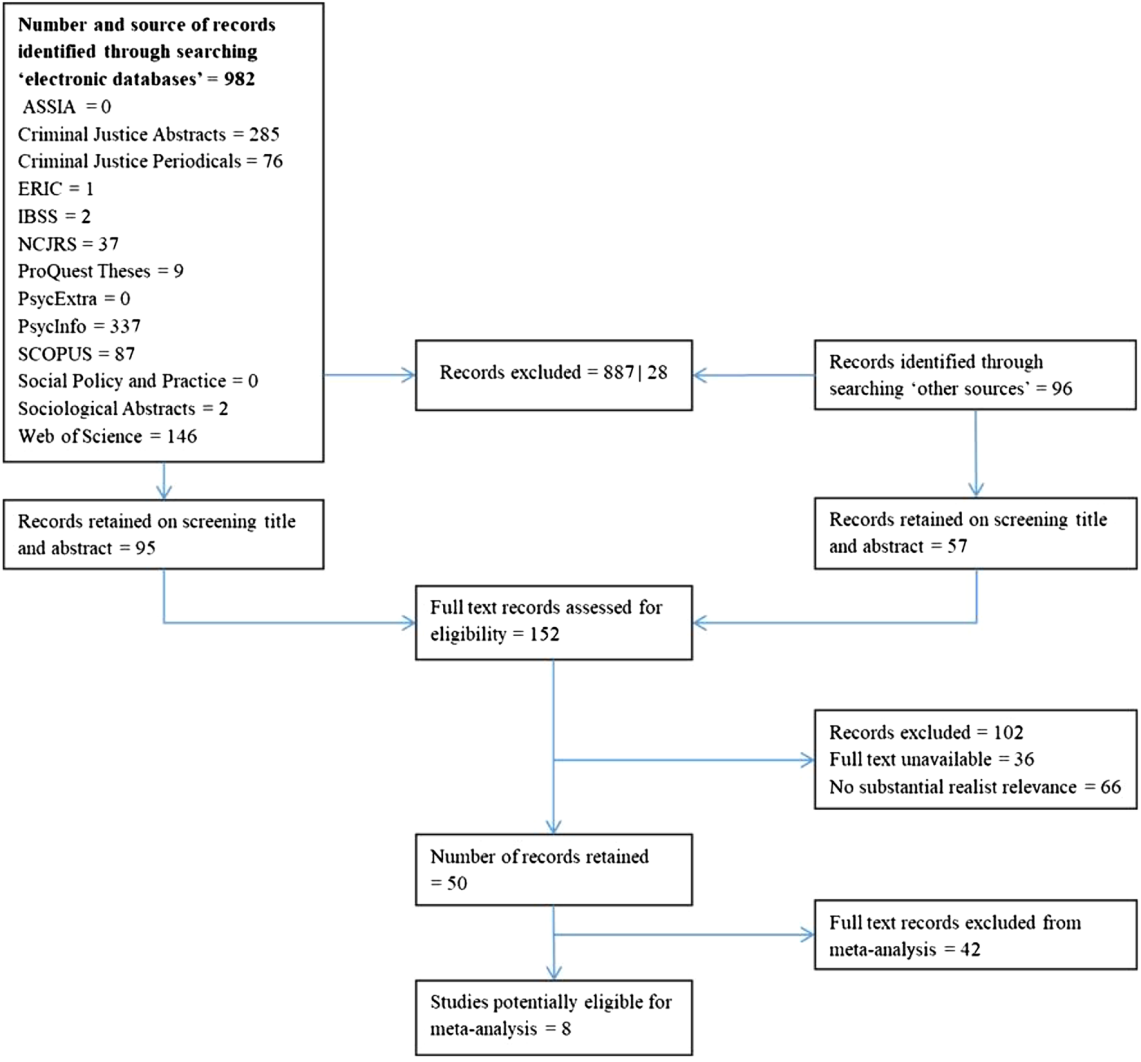
Flowchart of study selection

The number of, and reasons for, exclusions at each stage of the sifting process are shown in Fig. 1. In sum, fifty studies were judged relevant to tagging, all of which were analysed as part of our realist synthesis (see Additional file 1: Appendix 9 for a list of these studies). Of these fifty studies, eight made claims about the effectiveness of tags and were therefore deemed eligible for quantitative synthesis (“Effect” section).

It is noteworthy that our consultation with retailers produced four reports on tagging trials carried out by two retailers. In Fig. 1 these reports are included in ‘other sources’. Moreover, four retailers agreed to participate in semi-structured interviews on the use of tags and one retailer agreed to show members of the review team around a central London store to demonstrate how tags are applied in practice. All participating retailers asked that their identities remain anonymous and that the aforementioned industry reports not be shared.

## Effect

We identified eight studies that made causal inferences about the effectiveness of tags in retail environments. Characteristics of these studies are summarised in Table 1 and a narrative review is provided in Additional file 1: Appendix 10. Table 1 shows that five studies appeared in the scientific literature (journals or book chapters) and three studies were industry reports, two of which were conducted by a single retailer. Study dates ranged from 1993 to 2016. Seven studies examined the effectiveness of EAS tags and DiLonardo and Clarke (1996) was the only evaluation of ink tags. We found no evaluation studies of other types of security tag. All studies took place in either the USA (n = 4) or UK (n = 4), in supermarkets (n = 2), large retail stores (n = 3), predominately clothing stockists (n = 2) and a large electronics store (n = 1).

**Table 1:** Characteristics of studies with quantitative outcome measures studies included in “Effect” section

Study	Publication type	Location	Store type	Tag type	Item(s) tagged	Action group	Control group	Primary outcome measure(s)	Results
Farrington et al. (1993)	Book chapter	UK (country-wide)	Electrical goods stores	EAS tags	Electrical goods	2 stores with tags	1 store control, 2 stores other conditions (redesign and security guards)	% of items stolen	Significant long term decrease in number of items stolen in stores where tags were installed
Bamfield (1994)	Book chapter	UK (North and Midlands)	Variety chain retailer	EAS hard tags	All except those which cost <£5	4 stores	1 store	Shrinkage	28.3% reduction in shrinkage where EAS were installed
DiLonardo and Clarke (1996)	Journal	Hints at USA (country-wide)	Women’s clothing stores	Ink tags	Clothing	14 stores	None	Shortage	42% reduction in shrinkage where ink tags were installed
Hayes and Blackwood (2006)	Journal	USA (Michigan, Minnesota, Pennsylvania, New Jersey)	Mass merchant retail chain	EAS tags (concealed and not)	Personal grooming products	13 stores	8 stores	Item loss levels, product availability, sales	No significant difference in loss levels, product availability or sales figures
Beck and Palmer (2010)	Journal	USA (country-wide)	Apparel retailer (clothing, fragrances)	EAS hard tags vs EAS soft tags	Clothing	355 stores	540 stores	Shrinkage	250% increase in shrinkage following installation of soft tags
Downs et al. (2011)	Industry report	USA	Department store	A3 EAS tag (in red and beige) vs EAS tags	Jeans	3 stores	3 stores	Shrinkage and sales	Overall installation of A3Tags was associated with a 49% increases in shrinkage and a 5% increase in sales. However red A3Tags saw a 42% reduction in shrinkage and 18% increase in sales
Retailer A (2015)	Industry report	UK (country-wide)	Supermarket	EAS soft tags vs hard cases	CDs	20 stores	60 stores	Shrinkage, sales rates	134% increase in shrinkage following installation of soft tags; 16.6% increase in sales of tagged items compared to control stores.
Retailer B (2015)	Industry report	UK (country-wide)	Supermarket	EAS soft tags	Meat products	Number of stores not stated	Similar items in same store	Shrinkage	52.6% reduction in shrinkage following installation of EAS

Seven of the eight studies used some form of comparison group. This ranged from making comparisons between (1) similar but untagged products in the same store (Retailer B 2015), (2) different stores in which the specific tags under evaluation were not installed (Farrington et al. 1993; Bamfield 1994; Hayes and Blackwood 2006; Beck and Palmer 2010; Downs et al. 2011), and (3) the store chain average more generally (DiLonardo and Clarke 1996). The trial reported in Retailer A (2015) did use a comparison group but only in relation to changes in sales and availability. The impact of tags on shrinkage rates was assessed using a before and after design.

As shown in Table 1, there was considerable variation in the number of sites included in each study. For example, Farrington et al. (1993) reported on the effectiveness of EAS tags that were implemented in two stores compared to one store that was redesigned with security in mind, one store that received security guards and a ‘control’ store that received no additional security measures. Likewise, Bamfield (1994) examined a comparatively small sample of four action sites against one control site. The largest study was by Beck and Palmer (2010) which used data from a multibillion dollar US clothing retailer to examine the effects of switching from hard tags to source-tagged soft tags. Retailer B (2015) adopted a different approach to the other studies, whereby shrinkage levels for select lines of tagged meat products were compared to that of similar non-tagged items in the same store.

Although these eight studies all made causal inferences about the effectiveness of tags, on closer inspection we observed considerable heterogeneity across studies, particularly in terms of study outcome measures (discussed below). This was compounded by the different types of tags being evaluated (hard vs. soft EAS tags, visible vs. concealed tags) which, as we shall demonstrate, might plausibly give rise to different preventive mechanisms. We felt that these studies were too dissimilar to warrant a meaningful meta-analysis (see Petticrew and Roberts 2006, chapter 6). The sections that follow discuss the heterogeneity observed across these studies, looking first at study outcome measures and then at the findings of our risk of bias assessment. The third section draws some tentative conclusions about the effectiveness of tags based on a review of these studies.

### Heterogeneity in outcome measures

Table 1 shows that shrinkage/shortage was the most common primary outcome measure across the eight studies (n = 6). Additional outcome measures included sales rates and product availability. Commentators have long-observed variation in how shrinkage is conceived and measured (see Beck 2006, 2016a). Likewise in these studies, Bamfield (1994, p. 162) measured shrinkage as “the difference between actual sales + net stock compared with the previous period, and the book level of sales + stock”. DiLonardo and Clarke (1996) and Beck and Palmer (2010) both used store inventory statistics. Finally, Retailer A (2015) and Retailer B (2015) did not provide a clear definition of how shrinkage was measured, possibly for reasons of commercial sensitivity or simply because it was well-known internally. It should be clear that although each of these studies used some form of shrinkage, it is difficult to determine the comparability of these shrinkage estimates.

Our interviews with retailers revealed that the accuracy of the inventory counting processes that generate shrinkage estimates may vary both by business and product: fledgling businesses with less sophisticated delivery and tracking procedures may suffer a higher proportion of non-theft losses than more mature businesses with highly stringent, well-established systems in place; the delivery and tracking processes for high value items also tend to be more sophisticated than low value items. Moreover, from the perspective of *theft reduction*, an additional limitation is the inability to isolate the degree to which theft is a *source* of shrinkage, as opposed to other types of crime (such as fraud) and administrative errors. It is also likely to be unclear who perpetrated the theft—customers or employees (for a related discussion see Beck 2016a). It is worth mentioning that in some cases it appeared that the researchers had little influence over the data that were available to them. For example, Beck and Palmer (2010, p. 116) explicitly reported having “no control over the collection of the raw shrinkage data”. Similarly Downs et al. (2011, p. 14) add that they “had no control over the accuracy of the data provided by the participating retailer”.

It is noteworthy that we identified only two studies that included a *theft* outcome measure. In both cases collecting such data required considerable effort and resources on the part of the research team. Farrington et al. (1993) systematically counted the number of specified items on display each day. Shoplifting was inferred if the absence of a particular item could not be attributed to the item being sold, used, damaged, relocated or given away. It is important to add that this type of theft-specific information could not be gleaned retrospectively using inventory counting systems common to most retailers. Farrington et al. (1993) report that the research team was involved from the outset of the project and worked closely with the participating stores to provide training in and a rationale for this additional data collection procedure.

The second study reporting a theft outcome measure is Hayes and Blackwood (2006), who made use of various data including inventory counts and site observations. A novel feature of their study was the use of CCTV footage from selected stores to determine whether losses could be attributable to customer or employee theft.

### Risk of bias assessment

The risk of bias ratings for all eight studies is displayed in Table 2. Selection bias was found to be a methodological concern in all eight studies. No studies reported the use of inferential statistical tests to ensure equivalence of action and control groups before the installation of tags. Matching was typically based on similar store characteristics (such as size, layout, product range etc.) as opposed to outcome measures. Beck and Palmer (2010) display, but do not quantitatively assess, the volume and trajectory of shrinkage in action and control sites before the installation of tags. As mentioned above, Retailer A (2015) did not use a comparison group when assessing the impact of tags.

**Table 2:** Risk of bias assessment for eight studies included in “Effect” section

Study	Selection bias	Measurement bias	Regression to the mean	Contamination effects
Farrington et al. (1993)	Medium	Low	High	Medium
Bamfield (1994)	High	Medium	High	Medium
DiLonardo and Clarke (1996)	Medium	Medium	High	Medium
Hayes and Blackwood (2006)	Medium	Low	Low	Medium
Beck and Palmer (2010)	Medium	Medium	Medium	Medium
Downs et al. (2011)	High	High	High	High
Retailer A (2015)	Medium	High	High	High
Retailer B (2015)	High	High	High	High

Which stores received tags in some studies also raised concerns about representativeness. In Bamfield’s (1994) study, for example, tags were installed only in those stores that demonstrated a sufficient level of enthusiasm and successfully bid to receive the intervention. It is highly possible that successful store managers who are supportive of tagging are more likely to act in ways that might optimise tag effectiveness through, say, providing adequate training of staff, compared to store managers who were unsuccessful, failed to bid or were apathetic toward tags. Similar concerns about representativeness are apparent in DiLonardo and Clarke’s (1996) study, in which ink tags were installed in 14 newly opened stores and shortage levels compared to that of the storewide average. The authors acknowledge that although these two groups were considered comparable, a quantitative assessment of their equivalence was not possible given the data available.

Issues concerning potential measurement bias—the extent to which the data analysed were a reliable measure of theft—have already been covered. Farrington et al. (1993) and Hayes and Blackwood (2006) received favourable ratings because their outcome measures spoke more directly to theft. The three industry reports were deemed to be at high risk of bias since it was unclear how shrinkage was measured. Reasons for this are discussed briefly below.

To protect against regression to the mean effects (and confounding variables) studies implementing tagging in high-theft stores needed to be attentive to underlying trends in their data. Hayes and Blackwood (2006) was the only study to attempt to do this through triangulating data from multiple sources, and thus they received a low risk rating. Beck and Palmer (2010) used time series data to provide an indication of trends, but fell short of conducting a statistical test for seasonality or other patterns in their data, and hence were considered to be at medium risk of bias. In the remaining studies, either regression to the mean had not been taken into consideration or there was not enough information to judge. The five studies published in the scientific literature all readily acknowledged various potential confounds that could have affected the observed results. Regrettably, and likely owing to a lack of available data, none statistically examined the effect of these possible shortcomings, and hence received a medium risk rating for risk of contamination.

The three industry reports (Retailer A 2015; Retailer B 2015; Downs et al. 2011) warrant special mention. As seen in Table 2, based on the material presented, each trial received several high risk ratings. This was largely owing to insufficient information being provided on potential sources of bias. However, to some extent making comparisons between these reports and the aforementioned scientific articles is inappropriate. The reports made available to us were all short, pithy, and contained little superfluous information beyond the key priorities of retailers: what was done and what was found in relation to customer and staff reactions and, ultimately, sales. They were written for an internal audience who are likely to be familiar with how security devices are implemented and assessed in that particular business, and were likely presented with supplementary verbal accounts. They were not produced for external scrutiny on the research methods undertaken, as has occurred here.

### Overall findings of eligible tagging evaluations

What, then, can be said about the effectiveness of tags as a theft reduction measure in retail environments? Mindful of the aforementioned variability in outcome measures, if we assume that reductions in theft, shrinkage and shortage all denote positive outcomes associated with the introduction of tags, then across these eight studies we find mixed results. Considering all types of tags, five studies report positive results (Farrington et al. 1993; Bamfield 1994; DiLonardo and Clarke 1996; Downs et al. 2011 [specifically in relation to red Tags]; Retailer B 2015) (see Table 1). With the exception of Retailer B (2015), these studies all relate to the effectiveness of visible tags. Of these studies, Farrington et al. (1993) is unusual in collecting theft-specific data, finding that electronic tags produced significant and sustained reductions (over at least 6 weeks) in shoplifting compared to those stores where tags were not fitted. However, there are concerns over the representativeness of these findings considering the small number of stores that received tags (n = 2) and the limited time period over which tag effectiveness was assessed (1 week pre-intervention and up to 6 weeks post intervention).

As shown in Table 2, the methods used by Hayes and Blackwood (2006) are arguably the most robust of the eight evaluation studies we identified. Their quasi-experimental study related specifically to source-tagged concealed EAS tags affixed to personal grooming products. They found no significant differences in shrinkage, product availability or sales figures across test and control stores. By contrast, Beck and Palmer (2010) and Retailer A (2015) report an *increase* in shrinkage following the installation of tags. These apparent backfire effects warrant closer scrutiny. Beck and Palmer (2010), for example, assessed changes in shrinkage rates following the switch from more visible hard tags to less visible soft tags; it was not a conventional tag versus no tag evaluation. The resultant 251% increase in shrinkage in the action stores (compared to a 33% increase in shrinkage in control stores) may, therefore, be partly explained by the effectiveness of the previous (more visible) tag regime, consistent with the findings from other tag evaluations. As the study authors report, staff where the new tags were installed attributed the observed increase in shrinkage to “the lack of a visual deterrent to would-be thieves”, and as alarm activations increased, “staff members [became] less likely to respond [to sounding alarms] and more likely to simply wave customers through” (Beck and Palmer 2010, p. 119). Moreover, staff felt that the soft tags, once noticed by offenders, were easier to remove than hard tags, thereby bypassing the alarm system and further contributing to the increases in shrinkage. This hypothesis was based on an apparent increase in the number of discarded tags found in changing rooms. Also on the topic of tag visibility, Downs et al. (2011) showed that the installation of a new type of EAS tag in *red* produced reductions in shrinkage (42%) and increases in sales (18%) whereas for the beige counterpart, the reverse was true (producing a 252% increase in shrinkage and 7% decrease in sales).

The backfire effect reported by Retailer A (2015) also requires elaboration. As indicated in Table 1, this trial examined the impact of replacing secure casings for CDs with soft RF tags. The CD casings were considered too bulky and unattractive and were replaced with what were judged to be less obtrusive security measures. Shrinkage figures for tagged CDs were 134% greater over the 8-week trial period compared to the same time period before the tags were applied. Although clearly a negative result from the perspective of loss prevention, the authors report a corresponding *increase* in the sale of tagged CDs in 20 action stores (24.7%) compared to CD sales in 60 comparison stores where tags were not fitted (which saw an increase in sales of 6.3%), producing an overall net profit. Combined with reported improvements in the sale process and staff time (it was considered quicker and easier to deactivate the tags than remove the secure casings), the tag strategy was considered a success.

## Mechanisms

Mechanisms are taken here to refer to the processes through which tagging produces the observed effects (Pawson and Tilley 1997). It is important to acknowledge from the outset that none of the studies we identified contained a *quantitative assessment* of tag-related mechanisms nor did they report data that would allow for a retrospective analysis. Consequently, what follows is a descriptive account of the main mechanisms evident from the sources we scrutinised. Each is discussed here in isolation. In reality, however, it should be noted that tags might activate multiple mechanisms, giving rise to varying outcome patterns or working in concert to produce the same patterns jointly.

### References to mechanisms in the tagging literature reviewed

We assessed the prevalence of mechanism-related information in the 50 tagging studies we identified using a simple 3-point scale: (1) the study explicitly referred to how tagging is expected to work, (2) the study alluded to how tagging is expected to work, and (3) the study made no reference to the mechanisms through which tagging is expected to work. There are two obvious limitations with this method which warrant mention. First, we do not take account of the variation in the extent to which studies discuss mechanism-related information. Second, we do not make any judgements about the accuracy of the information relating to tag-mechanisms. For our purposes, we are simply interested in synthesising what the identified literature says about how tags may produce the outcomes observed.

Of the 50 studies consulted in the realist branch of our review, we judged that 27 (54%) included information regarding tag-related mechanisms (see Additional file 1: Appendix 11). Of those 27 studies, 18 explicitly referred to how tagging is expected to operate. This is a high proportion compared to other realist reviews of crime prevention interventions (see van der Knaap et al. 2008; Sidebottom et al. 2017). To illustrate, a study that we coded as alluding to tag-related mechanisms might refer to tags producing a deterrent effect. Farrington et al. (1993, p. 100), by contrast, *explicitly* made reference to mechanisms when they stated that “electronic tagging…[was] intended to have a deterrent effect by increasing the subjective probability of detection”.

We limit our focus here to those 27 studies that explicitly or otherwise reported information concerning tag-related mechanisms. What follows is a description of the three main mechanisms that emerged from these studies. As will become clear, certain mechanisms are associated with particular types of tags, and are assumed to work differently in different settings. The latter will be covered in more detail in the “Moderators” section.

### Increasing the risks

The dominant mechanism through which tagging is expected to work concerns increasing the risk of an offender being detected (referred to in 25 studies (50%), see Additional file 1: Appendix 11). Importantly, this mechanism can operate in two ways—either by altering the perception of risk or by influencing the probability of detection. To elaborate, tags might reduce theft because their presence discourages thieves from attempting to steal tagged items since their chance of detection is perceived to be elevated. In this scenario, thieves avoid *attempting* to steal tagged items. By contrast, the presence of tags may go unnoticed by offenders (particularly if the tags are concealed) or be spotted and ignored. In this scenario the offender proceeds to try to steal the item but the tag activates an alarm, which in turn mobilises staff and results in the offender being apprehended, thereby leading to reductions in theft. Hence the former refers to *perceived* risk, whereas the latter refers to *actual* risk of detection.

Both scenarios described above relate to increases in the risk of detection: the former serves to deter would-be thieves and the latter boosts the probability of an offender being apprehended. The latter is largely reserved for describing the effects of EAS tags. For non-electronic tags (such as ink tags), any associated increases in risk could only be produced should an offender attempt to remove the tag in store and be spotted by a member of staff (Bamfield 1992).

### Reducing the rewards/benefit denial

The second most frequently mentioned mechanism, referred to in six studies (12%) (see Additional file 1: Appendix 11) concerns the reductions in rewards or benefits brought about through using tags. In the studies identified, reward reductions were mainly discussed in relation to ink tags.^6^ Simply put, attempts to remove ink tags illegally might cause the tag to break, thereby releasing the ink and spoiling the sought after item. This in turn would presumably make the item less desirable and harder to sell.

### Increasing the effort

Gill et al. (1999), in their interviews with 38 shop thieves, discuss the topic of removing tags in store. This relates to a third albeit less frequently discussed mechanism through which tags might plausibly reduce theft: by increasing the effort required of offenders (mentioned in two studies). This mechanism might reduce theft in one of two ways. The first concerns the effort required to exit a store with a tagged item without raising suspicions of staff or other onlookers who might intervene. All things being equal, the required effort is likely to be higher for a tagged item than a non-tagged equivalent, most obviously in efforts to circumvent associated alarm systems (for EAS tags). A second way through which tags might increase offender effort relates to the actual removal of the tag, be that in-store or after the event. Again, it is plausible that thieves might be deterred from stealing products that require extensive efforts or tools to remove the tag. Although plausible, it should be noted that the literature we reviewed provided several examples of the methods and ease with which shoplifters were able to remove tags (see Bamfield 1994; Handford 1994; Farrington et al. 1993; Gill et al. 1999), thereby undermining this mechanism.

## Moderators

The terms ‘moderator’ and ‘context’ are used interchangeably in this section. They refer to the conditions that enable tags to activate potential causal mechanisms. Similar tags may, thus, activate different mechanisms depending on context, leading to variations in outcomes. As will become clear in the following two sections, some moderators are strongly influenced by the decisions and actions of those responsible for the implementation and management of tags, and so some of the same themes occur when discussing both moderators and implementation.

Twenty-eight studies (56%) contained information about moderators of tag effectiveness (see Additional file 1: Appendix 11). Eleven of these studies clearly stated one or more potential moderators and the other seventeen alluded to such influences. Taken together, these studies suggest that tagging and shop theft comprise a complex system, made up of interdependent individuals and organisations that adjust and adapt to one another. We identified five key elements that make up this system, all interacting in the causal processes at work in the operation of tags: (1) the shop (and its staff), (2) customers (including shoplifters), (3) tag technology (and its providers), (4) the product (and its designers), and (5) the police and criminal justice system. Discussing each in turn:

### 1. Staff responses and shop setting

All alarm systems are prey to false alarms, and the way in which staff and customers respond to these alarms is important (see Blackwood and Hayes 2003). Although on one hand, false alarms can be considered a negative consequence of EAS tagging, on the other hand they can also be viewed as a moderator of tag effectiveness. Regarding the latter, Beck (2002) finds that high false alarm rates (up to 93% in some cases) can reduce staff and shoplifter confidence in the alarms. For EAS tags, this can impede the aforementioned risk-elevating mechanisms. Hayes and Blackwood (2006) report only an 18% response rate to 4000 alarm activations, and even then staff usually failed to reconcile the items found on people with their till receipts.

False alarms have a range of sources including un-removed tags passing through the store gates, goods bought at other stores, untagged items that nevertheless trigger the alarm, and defects in the alarm system itself (Beck 2002). Failure to deactivate tags within store may be a function of either weaknesses in the system making deactivation problematic or lack of staff vigilance or training (Handford 1994). False alarms can cause embarrassment or anger to legitimate customers; some expect an apology and may be put off returning to the store in question, while others familiar with tags and their rationale have been found to be more understanding (Dawson 1993; Blackwood and Hayes 2006). False arrests resulting from false alarms have historically (and especially in the US) resulted in prosecution and reputational costs for the stores involved (see Bickman et al. 1979). Against the real risk of false alarms, Bamford (nd) suggests that where false alarms are occasional they may function as reminders to potential shoplifters that tags are being used in a store and thereby reinforce their deterrence value (increase risk mechanism).

Busy shopping periods compromise the scope for staff to respond to alarms. There is evidence of clear seasonal patterns to busyness, where greater busyness is associated with higher levels of shrinkage (Global Retail Theft Barometer 2015), although this shrinkage cannot be attributed to shoplifting alone. Physically the shop layout may facilitate or impede the operation of tagging systems. Doors without sensors offer an attractive low risk exit route for thieves with EAS-tagged goods. Moreover the space between gates affects the consistency of alarm activation (Huber 2006). The layout of the shop may offer greater or fewer opportunities for the shoplifter to remove tags inconspicuously within store and to walk out without triggering an alarm, and hence reduce risk of apprehension. The shop may or may not include signage that reminds customers of tagging (and other security measures) and/or CCTV systems that can be used in conjunction with tags to increase the perceived risk to shoplifters by supplementing the evidence that goods have been stolen (Beck and Palmer 2010; Capers 2008).

### 2. Type of shoplifter and customers

Two types of shoplifter are commonly referred to in the literature, with some empirical support for the distinction: the ‘casual’, ‘amateur’, ‘novice’, ‘impulsive’, or ‘opportunist’ and the ‘professional’, ‘hard core’ or ‘expert’ (Gill et al. 1999; Carmel-Gilfilen 2011; Hayes 1999; Beck 2002). Professional shoplifters tend to steal frequently, steal large quantities of goods, plan their shoplifting, steal for resale or refund, check stores for opportunities and risks, test the efficacy of security measures including tags, and work out ways of circumventing them. These methods of circumvention are clearly then disseminated, sometimes widely as is evident from readily available advice on the Internet. Casual shoplifters on the other hand tend not to plan shoplifting, steal for their own use or to give to others, and to take goods where opportunities manifestly present themselves. They may learn about opportunities to circumvent measures. They are less likely to develop them. For casual shoplifters, conspicuous tags with high levels of publicity are deemed effective and to deter thefts that would otherwise occur.

For professional shoplifters, deterrence is short-term and covert tags are deemed to have an effect through their scope to lead to arrests of shoplifters who are unaware of the risks they are taking (see Handford 1994; Capers 2008; Bickman et al. 1979; Lottes 1992). As described previously, Beck and Palmer’s (2010) observation that when hard, conspicuous tags were replaced with soft inconspicuous ones, losses increased dramatically, suggests that the visible deterrence that is of greater relevance to the casual shoplifter had been more effective, which may in turn suggest that casual shoplifters who are more easily deterred in this case were responsible for the bulk of the losses (see also Downs et al. 2011). Likewise, Buckle and Farrington (1984) in an observational study in a store in Peterborough (UK), which involved tracking and observing a random selection of 503 shoppers for an average of 6.9 min each found that 1.8% stole something and none was apprehended. In a more recent study from US, Dabney et al. (2004) found 8.5% of shoppers were observed shoplifting. These rates of shop theft suggest that many customers may be tempted occasionally to steal items.

### 3. Tagging strategy and technology

As mentioned previously, tags vary in their visibility; ‘soft’ tags tend to be inconspicuous and ‘hard’ tags conspicuous. The effect of conspicuous hard tags depends less on staff vigilance than soft inconspicuous tags in that they convey to the shoplifter the impression that they face *increased risks* of apprehension if they steal the goods. Soft tags may not be spotted by the shoplifter until the alarm sounds as they exit the shop and, if they are not stopped, then any potential crime prevention mechanism is undermined. Indeed, the tag may thereby become discredited as a source of increased risk in the eyes of the shoplifter (see Beck and Palmer 2010). In recent years several tag vendors have added symbols to soft tags in a bid to make them more conspicuous to potential offenders (Beck, personal communication).

Tagging dosage also varies. Of the 12 US-based retailers interviewed by Blackwood and Hayes (2003), seven were unsure of the proportion of merchandise that was (EAS) tagged and across the remaining five retailers the average was 26% of merchandise (ranging from 1 to 65%). In some stores there is a comprehensive tagging strategy. One retailer we visited as part of this study hard tagged all goods (except for shoes where only those for the right foot were on display). The tags used had features of EAS and ink tags, combining efforts in a bid to activate mechanisms associated with increases in perceived risk (EAS) and denying the benefits (ink tag) of shoplifting. This retailer also used an innovative method of attaching the tags to goods, which had (reportedly) yet to be circumvented by any shoplifters. Dramatic drops in shrinkage had been claimed by this retailer in the commercial press. The idea was to create stores that were comprehensively inhospitable to shoplifters.

Other tagging strategies include tagging frequently stolen goods, high value goods, implementing different types of tag (some of which may be decoy tags) and ‘fractional tagging’ whereby only a proportion of goods is ‘protected’ by tags in the expectation that diffusion of benefits effects (see Clarke and Weisburd 1994) will also reduce the rate at which untagged goods are stolen (Bender 1997; Masuda 1997; Hayes and Blackwood 2006). It is important to note that the opposite might also occur insofar that theft is displaced from tagged to untagged items (Bamfield 1994) or from stores with tags to stores without them (Farrington et al. 1993). No studies we identified revealed information on the difference these variations in tagging strategy had on the overall rate of shrinkage (studies did, however, examine the cost implications of fractional tagging, which we discuss in the “Economics” section).

### 4. Type of merchandise

The type of merchandise clearly shapes the type of tagging that is possible and the costs of applying it. Ink tags, for example, can quite easily be applied to clothes (DiLonardo and Clarke 1996) but are less relevant to other products. Meat, which is stolen in some grocery stores is not readily open to hard tags (Retailer B 2015). Some goods are so inexpensive that the costs of tagging would be prohibitive. Hence the potential for tagging and the activation of specific preventive mechanisms depends on a store’s product mix. Stores were found to consider the effect of tags on sales as well as theft in decisions about which tag to use and whether to use them at all. Some types of tag for some products make restocking more difficult and time consuming than others. For example, in one trial conspicuous bottle-top tagging of alcoholic drinks was found to make restocking more difficult than soft tags (Retailer C 2015). As discussed previously, tags were applied to CDs in one store because they made displaying and restocking more straightforward (than previous secure casing) and hence increased sales, even at the expense of in-retailer research that showed there were more thefts of them (Retailer A 2015).

### 5. Police and criminal justice system

Depending upon whether the goal of the retailer is to deter theft or detect and apprehend offenders, the response of criminal justice agencies (responsible for arrest, prosecution, conviction and punishment) is important. This concerns not only the decisions agencies take but also the speed with which they (and in particular the police) react. This in turn feeds back into the tagging strategies adopted. One UK retailer we interviewed remarked that it often took the police over an hour to come to a shop if it reported that a suspected shoplifter had been detained. This created three problems. First, detaining someone, if they are violent, creates risks for store personnel. Second, at least two people are taken off the shop floor whilst the person is held. Third, there could be no certainty that a person who was detained would eventually be charged, prosecuted and convicted. For these reasons, the store elected only to detain offenders in extreme circumstances, that is when they had provided a prior warning to the individual, when they had provided a visible presence when that person was within a store (they had a high quality CCTV system), and when nevertheless the person still attempted to steal goods as they left the shop. This happened infrequently.

## Implementation

Problems of implementation are a common feature of situational crime prevention (Knutsson and Clarke 2006). In the context of this review, implementation refers to the practical task of installing tags so as to optimise the conditions for them to work effectively. Put differently, those actions that best ensure the *context* is sufficient to activate the sought after preventive mechanisms. Of the 50 studies we identified, 36 mentioned implementation issues and of those, 29 contained detailed information on specific aspects of implementation (see Additional file 1: Appendix 11). In what follows this information is organised into two main themes: staffing issues and choice of tagging strategy.

### 1. Staffing issues

Store staff clearly play an important role in the installation and management of tags. Many of the aforementioned moderators of tag effectiveness relate to the decisions and actions of staff. There were several examples of implementation failures that were attributable to staffing problems. These included failure to correctly attach tags (Farrington et al. 1993), double tagging (Handford 1994; Huber 2006), or attaching tags so that they cannot be easily removed (Bamfield 1992; Beck 2006; Beck and Palmer 2010). Moreover, studies found that tags were often not deactivated properly (Handford 1994; Beck 2002) or that staff failed to react appropriately to activated alarms (Baumer and Rosenbaum 1984; Blackwood and Hayes 2003). Maximising the probability that tags are appropriately and consistently attached to items, that they are properly removed or deactivated at point of sale, or that sounding alarms are responded to—moderators of tag effectiveness related to implementation—was generally considered to be influenced by the extent to which staff are adequately trained, monitored and incentivised to participate in a tagging programme.

### 2. Tagging strategy

Decisions about the type of tag(s) to use are obviously dependent on cost (discussed in “Economics”), store design and the items intended for tagging. In addition, retailers must decide on an appropriate tagging strategy. This can take several forms:

- *Source tagging vs. in*-*store tagging* As already mentioned, source tagging involves a tag being incorporated into the label, fabric or packaging of a product at the point of manufacture. Here, specialised staff or processes at point of manufacture can ensure the proper and consistent application of tags rather than store staff, who have to be trained and resourced to do so. Source tagging is thus often preferable to (and increasingly adopted by) retailers since it absolves them from having to tag items in store (Beck and Palmer 2010). However, a major difficulty for source tagging is that different manufactures (and retailers) often use different types of tags (for e.g., AM vs. RF EAS tags), each requiring corresponding detection and removal equipment (Beck 2002). This is a particular problem for stores selling products supplied by different manufacturers and potentially different tagging systems.
- *Universal tagging vs. fractional tagging* We have already seen in the “Moderators” section how tag dosage is thought to affect offender perceptions. Yet how tags are applied to products is also a practical decision. Universal tagging is resource intensive and can be expensive. However, whether it is preferable to tag fractionally or to tag selectively only some expensive or desirable products will depend on the type of merchandise being sold and on the risk of shop theft given the shop’s location and type of clientele.

## Economics

Thirty-two of the 50 studies (64%) contained economic information relating to tagging. A narrative summary of this information is provided here, both in terms of the cost *and* cost effectiveness of tagging. Despite the high prevalence of economic information in these studies, regrettably this information was not sufficient to conduct a full economic evaluation (such as cost-benefit analysis).

### Cost of tagging

The cost of tags was found to vary widely across studies. This was mostly attributed to the type of tag and their re-usability. For example, disposable RF EAS tags are now available for as little as a penny each (Loebbecke and Palmer 2006). EAS reusable tags cost around 20–35p (Retailer D 2015). The most expensive tags (in terms of initial outlay) appear to be ink tags, which are designed to be reusable. However, ink tags typically require less infrastructure and therefore have lower set-up costs than EAS tags since they do not require electronic gates.

Information on the costs of the wider EAS tag system (electronic gates at store entrances and exits, de-tagging machinery, tag readers) received less coverage in the studies we identified (n = 15). These studies all alluded to retailers having to account for more than the costs of tags when deciding upon a system. For example, a large retailer must implement the same system across many stores. Conversations with retailers have suggested that £2000 for a present-day de-tagging device would not be uncommon, and stores will often have multiple de-tagging devices.

A further set of costs relate to employees, whether this is hiring new security guards to monitor electronic gates, training existing staff to handle new tagging systems, or the hours required to tag and de-tag products in store (if adopting this type of tagging strategy). Studies from retailers suggest that these costs are keenly observed as part of tagging trials. Two trials conducted by major retailers, one of soft RF EAS tags on CDs and another of magnetic tags on wallets, measured the amount of time in seconds taken to apply and remove the tags (12 and 14 s, respectively; Retailer A 2015; Retailer D 2015). These figures were then converted into an estimate of the annual number of staff hours required should the tags be rolled out across all stores (around 25,000 h in Retailer A), based on the predicted volume of CDs and wallets (in the several millions for both items). These calculations produced monetary estimates which were then considered as part of the overall performance of installed tags.

### Economic returns associated with tagging

Considerations over the economic returns associated with tagging relate to the various roles which tags are expected to play in retail environments including loss reduction but others too (such as stock tracking and management). As alluded to previously, there is also the issue of whether reductions in shrinkage generate an uptick in sales, and whether this can be reliably attributed to the use of tags (presumably through increased stock availability), as was found in the evaluation of red EAS tags by Downs et al. (2011). These wider benefits consequent on tagging create complications when attempting to conduct a thorough cost-benefit analysis. (for a related discussion see Beck 2008; Chainlink 2014). Notwithstanding the obvious importance of assessing cost-effectiveness, in their interviews with a convenience sample of 12 US retailers, Blackwood and Hayes (2003) found only a quarter carried out routine assessments of the return on investment following the installation of tags. Such assessments are, however, particularly important for small retailers, where even low levels of shrinkage can have significant negative effects on profit margins (DiLonardo 1996).

We have already mentioned the different types of tagging strategy available. The tagging strategy employed by retailers has cost implications. One way in which retailers may reduce their expenditure is to apply tags in their own supply chain (Beck, personal communication). An additional approach is to work with a supplier who tags items at source, rather than to apply tags in-store (Beck and Palmer 2010). However, this may lead to the cost of tagging being forced upon manufacturers instead, many of whom may be reluctant to absorb such costs (Chainlink 2014). There are examples in the literature of manufacturers being persuaded by large retailers to apply tags. Retailers use various methods, including threats no longer to stock the product, promises of increases in sales and shelf space, and offers to share the costs of tagging. Equally important to note is that, if tags are effective, then if manufacturers agree to apply them at source, a boost in sales should ensue, benefitting manufacturers and retailers alike.

Tagging at source can also assist ‘fractional tagging’ and the sought-after ‘halo effect’ (or diffusion of benefits) of tagged items providing protection to non-tagged items. The economic implications of such a ‘halo effect’ were quantified by one large retailer who saw savings of tens of thousands of pounds in reduced shrinkage amongst similar non-tagged items, which was included in the cost-benefit analysis of the tagging trial (Retailer C 2015). Another study found this effect moved to unrelated items within the store (Masuda 1997). This potential diffusion of benefits could be quantified by researchers and used to rank some systems over others, by saving money and increasing margins.

## Discussion

Tags are commonly used in retail environments, but their effectiveness as a theft reduction measure has yet to be the subject of a systematic review. In this paper we followed the EMMIE framework (Johnson et al. 2015) to review the evidence as it relates to (1) whether tags are effective at reducing theft, (2) the causal mechanisms through which tags are thought to work, (3) the contextual factors that moderate tag effectiveness, (4) how tags are implemented in retail settings and (5) the economics of tagging.

Following a systematic search of the published and unpublished literature, and through consultation with retailers, we identified fifty studies that met our eligibility criteria. Eight studies reported quantitative data and were assessed for information concerning the effectiveness of tagging. On closer scrutiny, substantial variation in the type of tag installed and how tag effectiveness was measured precluded a meta-analysis. Concerns about selection bias were also noted since no evaluation study reported any statistical analyses to determine the equivalence of action and control groups before intervention. Drawing firm conclusions about the effectiveness of specific types of tag is therefore challenging. For example, we found only one study on the effectiveness of ink tags, and that dates back some 20 years (DiLonardo and Clarke 1996). Likewise with EAS tags, whilst several early studies converge on the finding that tagging is effective (Farrington et al. 1993; Bamfield 1994), evidence from a larger and more recent study with a stronger research design found tagging to have no noticeable impact (Hayes and Blackwood 2006). Moreover, studies such as that by Beck and Palmer (2010) speak more to the comparative effectiveness of different forms of EAS tag (hard tags vs. soft tags) than to the effectiveness of tagging per se. Despite this variation, across the eight evaluation studies we identified, evidence does suggest that more visible tags tend to be associated with greater reductions in shrinkage than less visible tags.

The complexity of tagging was further elucidated through the realist branch of our review, which examined a wider range of studies supplemented with interviews with four retailers. It is clear that different types of tag are expected to produce reductions in theft through different *mechanisms,* which in turn require contrasting conditions for their activation (*moderators*), and which give rise to different *implementation* challenges. EAS tags, for example, are widely assumed to reduce theft through increasing the (perceived or actual) risk that offenders are apprehended. Activation of these risk-enhancing mechanisms is in turn influenced by factors such as tag visibility (did offenders spot the tag?), staff behaviour (did staff respond to the sounding alarm?) and the type of shoplifter thought to operate in store (were offenders deterred by the tagging system?). Ink tags, by contrast, are generally assumed to reduce theft because of the inconvenience associated with removing the tag and the potential release of indelible ink, thereby spoiling the product and making it harder to sell. This variation in how different tags are expected to reduce theft also suggests that pooling information across tag types (in, say, a meta-analysis) to generate an overall conclusion is inappropriate.

It is noteworthy that we identified no high-quality published economic evaluations of tagging (i.e., estimates on the direct and indirect economic costs and benefits of a tagging strategy). Although economic analysis remains infrequent in the crime prevention literature more generally (see Manning et al. 2016), its absence in the context of this review is surprising given the high priority retailers place on cost effectiveness. We suspect this lack of economic evaluation is a product of data *accessibility* rather than data *availability*. Consultation with retailers in the UK as part of this review indicated that economic data are available and that trials on the cost effectiveness of tags are routinely undertaken, albeit that the results of such trials are seldom made public for commercially sensitive reasons. However, it is difficult to determine how representative such actions are, especially given evidence from a convenience sample of 12 US-based retailers suggesting that robust cost-benefit analysis of tagging remains infrequent (Blackwood and Hayes 2003). Further research is needed to determine the range of financial costs and outcomes associated with tagging, and how these vary by tag type and product.

### Implications for practice and research

In reviewing the literature on tagging we identified several topics where future research might usefully be directed. The first knowledge gap concerns crime displacement/diffusion of benefits (Guerette and Bowers 2009) associated with tagging, which was alluded to in several studies (Farrington et al. 1993; Beck and Palmer 2010) but not empirically examined. Moreover sufficient data were not reported for displacement to be analysed retrospectively by the review authors. The closest formal assessment was provided in two retailer reports which sought to quantify the economic impact of “halo effects” on related but non-tagged products (Retailer B 2015; Retailer C 2015). In the context of tagging in retail environments, crime displacement/diffusion of benefits could take several forms: (1) target displacement/diffusion of benefits *within* stores from tagged to non-tagged items, (2) spatial displacement/diffusion of benefits to nearby *different* stores and (3) spatial displacement/diffusion of benefits to stores of the same chain (where applicable) located *elsewhere*. This is an area where future research might usefully be directed, not least because interviews with shoplifters conducted by Giblin et al. (2015) revealed that a small proportion reportedly would look to shoplift elsewhere upon confronting a perceived credible tagging strategy.

Displacement usually refers to the actions of *individual* offenders. Adaptation refers to the longer-term process of *populations* of offenders seeking to overcome situational measures (Clarke and Bowers 2017). The literature we reviewed contained several references to the many ways in which offenders tried to bypass or override tagging systems (Handford 1994; Farrington et al. 1993). Despite this, we identified little evidence on the long-term effectiveness of tags. Addressing this gap is important given (1) the changing nature of retailing in general (such as the introduction of self-service checkouts) and tagging in particular (such as the introduction of new types of tags), (2) the noted adaptive and innovative capacity of shop thieves in response to prevention measures; and (3) the aforementioned challenges of sustaining a tagging strategy where tags might variously be dysfunctional, damaged or disappear. The longest study period of an evaluation study we identified was 12 months post intervention (Beck and Palmer 2010). Future research might usefully investigate the sustainability of any preventive effects associated with tagging, both to explore the scope for offender adaptation and the practical task of maintaining an effective tag system over time.

## Additional file

**Additional file 1.** Appendices.

## Footnotes

1 This cost included theft by employees and the cost of fraudulent suppliers, as well as shoplifting and the cost of loss prevention systems.

2 Tags are not used solely for the purposes of theft reduction, however. They are also implemented for the purposes of product authentication (to detect counterfeit items) and as a means of assisting supply chain management. Nor is their use limited to retail environments. EAS tags are used in airports to track the movement of baggage (Mishra and Mishra 2010), in hospitals to track new-borns and elderly patients and in prisons to monitor inmates (Hickman et al. 2010).

3 All retail-related journals were found to be covered by the two multidisciplinary databases (SCOPUS and Web of Knowledge).

4 These are *Police Practice and Research: An International Journal* and *Policing: a Journal of Policy and Practice.*

5 Due to limited resources, we confined our forward and backward citation searches only to these “Effect” studies.

6 Bottle tags are also assumed to work through this mechanism. These refer to hard tags that are placed over the neck of bottled products (typically alcoholic beverages). Failure to correctly remove the tag causes the bottle to break.
