# Supplementary material for: A systematic review of tagging as a method to reduce theft in retail environments
Source: Crime Sci. 2017 May 30;6(1):7. doi: 10.1186/s40163-017-0068-y (PMC6979536; doi:10.1186/s40163-017-0068-y)
Supplement: Supplementary file 1 — Additional file 1. Appendices. [file 40163_2017_68_MOESM1_ESM.docx]

# APPENDICES

## Appendix 1. Keyword search terms for electronic databases

We used the following terms when searching the electronic databases. Where necessary, search terms were adapted to suit the requirements of particular databases:

TITLE-ABS-KEY (shoplift* OR ((shop OR store OR retail OR customer OR employee ) W/5 ( loss* OR shrinkage OR crim* OR theft OR steal OR stolen) ) AND ( tag* OR eas OR "Electronic theft control system*" OR "Electronic article surveillance" OR "Electronic asset surveillance" OR "Acousto-Magnetic" OR "radio frequency") )

Searches were conducted on 23^rd^ September 2015.

## Appendix 2: Electronic and grey literature databases searched in this review

1) ASSIA (Applied Social Sciences Index and Abstracts)

2) Criminal Justice Abstracts

3) Criminal Justice Periodicals

4) ERIC (Education Resources Information Center)

5) IBSS (International Bibliography of Social Sciences)

6) NCJRS (National Criminal Justice Reference Service)

7) ProQuest theses and dissertations

8) PsycINFO

9) PsycEXTRA

10) SCOPUS

11) Social Policy and Practice

12) Sociological Abstracts

13) Web of Science

14) CINCH (Australian Criminology Database)

## Appendix 3: Retail journals searched in this review

International Journal of Retail & Distribution Management

International Review of Retail, Distribution and Consumer Research

Journal of Retail and Leisure Property

Journal of Business and Retail Management Research

European Retail Research

Journal of Applied Security Research

Security Journal

Journal of Retailing

Journal of Retailing and Consumer Services

Retail Security and Loss Prevention

Retailing in Emerging Markets: A Policy and Strategy Perspective

## **Appendix 4: Keyword s**earch terms for ‘other sources’ searched in this review

We used the following terms when searching the ‘other sources’ listed below:

1. Anti-theft tags
2. Anti-Theft solution*
3. Source-tagging
4. EAS tags
5. Ink tags
6. Electronic Article Surveillance
7. Radio Tags

## **Appendix 5**: Other sources searched in this review

In collaboration with an information specialist (Phyllis Schultze, Rutgers University) we searched the publications of the following government, research and professional agencies:

1) Center for Problem-Oriented Policing (Tilley and Goldstein Award entries)

2) Institute for Law and Justice

3) Vera Institute for Justice (crime and victimization publications)

4) Rand Corporation (public safety publications)

5) Police Foundation

6) Police Executive Research Forum

7) The Campbell Collaboration reviews and protocols

8) Urban Institute

9) European Crime Prevention Network

10) Swedish National Council for Crime Prevention

11) UK Home Office

12) UK College of Policing (Polka)

13) Australian Institute of Criminology

14) Swedish Police Service

15) Norwegian Ministry of Justice

16) Canadian Police College

17) Finnish Police (Polsi)

18) Danish National Police (Politi)

19) The Netherlands Police (Politie)

20) New Zealand Police

21) US National Institute of Justice

We also searched:

1) Google

2) Google Scholar

3) Academic Search Premier (EBSC)

4) ProQuest Sociology

5) Rutgers Criminal Justice Grey Literature Database

6) OSCE Polis Digital Library

7) WorldCat

## **Appendix 6:** Experts consulted in this review

Joshua Bamfield, Adrian Beck, Robert DiLonardo, Martin Gill and Read Hayes.

## **Appendix 7:** **Copy of e-letter sent to retailers by the *Metropolitan Police Service Business Crime Hub***

Dear Business colleague,

The MPS Business Crime Hub would be grateful for your assistance with a piece of important crime reduction research on behalf of the College of Policing which is being led by Dr Aiden Sidebottom (University College London).

Researchers from University College London are conducting a review on the effectiveness of security tagging (broadly defined) as a means of preventing retail theft. The aim is to determine what is known about how and where security tagging can be used effectively to prevent retail theft, findings which clearly will be of relevance to retailers and the police. This research is jointly funded by a major UK Research Council the College of Policing supporting the What Works Centre for Crime Reduction.

The research team is currently assembling the available evidence on whether tagging items has been effective as a method of reducing theft. The more evidence they assemble, the greater the confidence that can be placed in the overall findings. To that aim, they are keen to hear from retailers who have undertaken trials to establish the effectiveness of tagging items, mindful that the findings of such trails may not have been published. The hope is that any relevant findings can be incorporated into the overall analysis.

Please be reassured that information received from businesses will be kept in confidence - Names of specific retailers or products will not be mentioned in any outputs of this review.

The Business Crime Hub strongly supports this research. We therefore ask those who are aware of any trials of the effectiveness of product tags or if you are willing to assist in this research, might kindly contact Dr Aiden Sidebottom who is leading this review.

For more information please find attached a copy of the review protocol, which sets out in greater detail the aims and methods of the project, or feel free to contact Dr Aiden Sidebottom directly.

## Appendix 8: Information extracted from included studies

1. Study details (title, year, author(s), publication status, study location(s))
2. Type of tag in study (e.g. RFID, ink, magnetic, visible vs. invisible)
3. Total number of tags fitted (intensity)
4. Proportion of merchandise tagged
5. Type of merchandise tagged
6. Where the tags were implemented (at source or in-store?)
7. Type of retail environment (department store; supermarket; warehouse retailers; speciality retailers; convenience retailers; discounter retailer)
8. Details on retail environment (size of store, products sold, location, crime history)
9. Unit of analysis (e.g. store, chain of stores)
10. Research design (RCT, quasi-experimental)
11. Description of comparison group, place or period
12. Sample (size and any notable features)
13. Statistical test(s) used
14. Outcome measure reported and data source
15. Other interventions implemented over the study period including routine security practices - for example the employment of private security guards.
16. Indirect effects (displacement and/or diffusion of crime control benefits)
17. Information concerning the possible mechanisms through which tags could reduce theft
18. Information concerning the possible moderators that influence tag effectiveness
19. Information concerning the implementation of tags
20. Financial costs and benefits of tags as a theft prevention measure
21. Conclusions of the author(s)

## Appendix 9: Studies included in this review

| **Study author(s)** | **Study title** | **Date** |
| --- | --- | --- |
| Addis, K., Arbetter, L., Murphy, J. | Better red than stolen | 1993 |
| Addis, K., Arbetter, L., Harowitz, S., Murphy, J., Wilson, C. | Going to market | 1993 |
| Anonymous | Small tags protect big stores | 1982 |
| Bamfield, J. | EAS: management learning in curbing theft | 1997 |
| Bamfield, J. | Changing Retail, Changing Loss Prevention | c.2012 |
| Bamfield, J. | Beating the Thief: A Retailer’s Guide to Electronic Article Surveillance | 1992 |
| Barua, A., Mani, D., Whinston, A. | Assessing the Financial impacts of RFID Technologies on the Retail and Healthcare Sectors | 2008 |
| Baumer, T. and Rosenbaum, D. | Combating Retail Theft: Programs and Strategies | 1984 |
| Beck, A. and Palmer, W. | The Importance of Visual Situational Cues and Difficulty of Removal in Creating Deterrence: The Limitations of Electronic Article Surveillance Source Tagging in the Retail Environment | 2010 |
| Beck, A. | Automatic Product Identification & Shrinkage: Scoping the Potential | 2002 |
| Beck, A. | The Emperor Has No Clothes: What Future Role for Technology in Reducing Retail Shrinkage? | 2007 |
| Beck, A. | Preventing Retail Shrinkage: Measuring the ‘Value’ of CCTV, EAS and Data Mining Tools | 2008 |
| Beck, A. | Shrinkage and RFID: prospects, problems and practicalities | 2006 |
| Bender, W. | Playing tag is serious business. Security Management | 1997 |
| Bickman, L., Rosenbaum, D., Baumer, T., Kudel, M., Christenholz, C., Knight, S., Perkowitz, W., Everett, M., Smith-Bickman, L. | National Evaluation Program - Phase I Assessment of Shoplifting and Employee Theft Programs - Summary Report | 1980 |
| Blackwood, R. and Hayes, R. | EAS Retail Theft Protection Systems: An Issue Update | 2003 |
| Blackwood, R. and Hayes, R. | Shoplifting Solutions Survey  2006 RILA Loss Prevention Conference Survey | 2006 |
| Capers, C. | Effectiveness of situational crime prevention strategies to deter organized retail theft | 2008 |
| Carmel-Gilfilen, C. | Bridging security and good design: Understanding perceptions of expert and novice shoplifters | 2013 |
| ChainLink Research | The ROI for RFID in Retail: Use Cases Driving the Current Surge in RFID Adoption | 2014 |
| Conrad, A. | 'Smart' apparel | 1996 |
| Dawson, S. | Consumer responses to electronic article surveillance alarms | 1993 |
| Dilonardo, R. | Defining and measuring the economic benefit of electronic article assessment | 1996 |
| Dilonardo, R. and Clarke, R. | Reducing the rewards of shoplifting: an evaluation of ink tags | 1996 |
| DiLonardo, R. | Electronic article surveillance | 2008 |
| Downs, D., Hayes, R. and Tallman, C. | Product Protection Research Project: A3Tag – Testing the Effectivenss of the A3Tag on Sales and Loss | 2011 |
| Farrington, D., Bowen, S., Buckle, A., Burns-Howell, T., Burrows, J., Speed, M. | An Experiment on the Prevention of Shoplifting | 1993 |
| Giblin, M.B., Grottini, K. and Hayes, R. | Discount Apparel Mix Tag Offender Interview Report | 2015 |
| Gill, M., Bilby, C., Turbin, V. | Retail Security: Understanding What Deters Shop Thieves. | 1999 |
| Gohil, M. and Trikha, M. | RFID Used for Anti-Theft in Shopping Malls: A Research Survey | 2013 |
| Gorshe, M., Rollman, M., Beverly, R. | Item-level RFID: A Competitive Differentiator | 2012 |
| Handford, M. | Electronic tagging in action: a case study in retailing | 1994 |
| Hayes, R. and Blackwood, R. | Evaluating the Effects of EAS on Product Sales and Loss: Results of a Large-Scale Field Experiment | 2006 |
| Hayes, R. | Tailoring Security to Fit the Criminal | 1999 |
| Hayes, R. | Retail Crime Control: An Operational Strategy | Unknown |
| Hayes, R. and Cordone, C. | Shoptheft | 2006 |
| Huber, N. | Minimizing product shrinkage in the supply chain through the use of radio-frequency identification: A case study on a major Australian retailer | 2006 |
| Jones, P., Clarke-Hill, C., Hillier, D., Comfort, D. | The benefits, challenges and impacts of radio frequency identification technology (RFID) for retailers in the UK | 2005 |
| Klein, F. | The World's Largest Cookie Jar | 1982 |
| Lindblom, A., Kajalo, S. | The use and effectiveness of formal and informal surveillance in reducing shoplifting: A survey in Sweden, Norway and Finland | 2011 |
| Loebbecke, C. and Palmer, J. | A Real-World Pilot of RFID along the Fashion Industry: Kaufhof and Gerry Weber in Germany. | 2006 |
| Longmore-Etheridge, A. | The evolution of EAS | 1998 |
| Lottes, S. | What's in Store with EAS | 1992 |
| Masuda, B | Reduction of Employee Theft in a Retail Environment: Displacement vs Diffusion of Benefits | 1997 |
| Patton, J. | RFID as electronic article surveillance: Feasibility assessment | 2008 |
| Sensormatic | Electronic Article Surveillance: A Technology Comparison | Unknown |
| Retailer A, 2015 | Soft tagging CDs trial | 2015 |
| Retailer B, 2015 | Meat, Fish & Poultry RF Tagging | 2015 |
| Retailer C, 2015 | Alcohol RF tagging | 2015 |
| Retailer D, 2015 | Wallets trial | 2015 |

## Appendix 10: Narrative review of the seven studies included in Effect section

***Electronic Article Surveillance: management learning in curbing theft*** *-* Bamfield (1994)

Bamfield describes an evaluation of a pilot trial of EAS hard tags in a (predominantly) clothing and textiles chain retailer in the North and Midlands of England. The four treatment stores were selected on the strength of their managers’ plan for implementing and monitoring the intervention. The (single) control store was chosen to be similar in location, size and turnover to one of the four treatment stores. Hard tags were implemented for six months, with security guards and detector gates installed at the sole entrance/exit point. 90 per cent of merchandise was tagged with hard tags (with complete coverage for each product line). Merchandise under £5 and ‘hard’ goods, such as music cassettes, were not tagged. Bamfield presents changes in shrinkage rates alongside qualitative information on levels of in-store aggression and violence over the intervention period, how the EAS tags were perceived by the retailer, whether displacement occurred, and the cost-effectiveness of the tags.

***The Importance of Visual Situational Cues and Difficulty of Removal in Creating Deterrence: The Limitations of Electronic Article Surveillance Source Tagging in the Retail Environment*** *-* Beck and Palmer (2010)

The experiment reported by Beck and Palmer concerned the effect of a retailer switching from using ‘hard’ tags applied in store by staff (e.g. EAS) to source-applied ‘soft’ tags (acousto-magnetic technology). The U.S. retailer sold clothing, fragrance and belts and had a countrywide presence. The study, conducted from January 2006 to September 2007, trialled soft tags being sewn into garments during the manufacturing process in 355 stores of one branded outlet. Two other branded outlets, comprising 540 stores similar in size and location, acted as the control group. Additional staff and CCTV were provided in experimental stores deemed to be most at risk of shrinkage. The proportion of shrinkage was compared across experimental and control stores for six data capture points at four-month intervals. When it became apparent that shrinkage was increasing markedly in the experimental group, the retailer reintroduced hard tags. Beck and Palmer identified that the number of alarm activations rose considerably in experimental stores during the trial, and staff were less likely to respond in the wake of so many alarms being activated. Employee theft was also believed to have increased during the trial period.

***Reducing the rewards of shoplifting: An evaluation of ink tags*** - DiLonardo and Clarke (1996)

The two small case studies presented by DiLonardo and Clarke evaluate the implementation of ink tags in a large chain of women’s clothing stores with different branded outlets. The first involved 14 newly-opened, and geographically dispersed, stores whereby ink tags were installed as standard from opening. Physical inventories of stock were undertaken in the first year of opening and compared against the other stores in the chain. The second case study concerned the replacement of EAS with ink tags in four stores with the highest shrinkage rates.

***Product Protection Research Project: A3Tag – Testing the Effectivenss of the A3Tag on Sales and Loss*** – Downs, Hayes and Tallman (2011).

Using data from a large department chain in the US, this study assessed changes in the shrinkage and sales of jeans following the installation of a new type of EAS tag known as the A3Tag. Six studies participated in this study. In two stores, a beige version of the new A3tag was installed. In a third store a red version of the A3tag was installed, and three stores acted as control sites in which existing EAS tags were in place. Downs et al. report that overall there was little difference in shrinkage and sales rates in the treatment stores compared to the control stores (both saw increases in shrinkage). However, when examining the effectiveness of the different type of A3Tag in isolation, it was found that the more visible red tag was associated with reductions in shrinkage and increases in sales compared to the control store (a positive outcome). For the beige A3tag the reverse was true (increases in shrinkage and decreases in sales – a negative outcome, but one that mirrored the trend in control stores, possibly due to a seasonal effect). This difference in effectiveness was mainly attributed to the increased visibility of the red tags. Interviews with a sample of customers and employees indicated that the new type of tag was generally well received.

***An experiment on the prevention of shoplifting*** – Farrington et al. (1996)

Farrington and colleagues describe an experiment to evaluate the effectiveness of EAS tags, store redesign and security guards in a U.K. electrical and electronics retailer, across two branded outlets. A previous study by the authors had measured the shoplifting rates at twenty-nine stores across the U.K. with management trainees performing the repeated, systematic counting of items on store shelves. The ten stores with the highest shoplifting rates were chosen for the study; but the attrition of one store (due to heavy snow) reduced this number to nine. Comparable stores were assigned into four treatment groups, with three of these groups containing a control store. The other treatment group compared tags with store redesign. Staff were trained in the measurement technique used previously by the authors, with visits post-treatment to check that this was effectively undertaken. Audiotapes, videotapes, films, headphones and small domestic appliances were the product lines under scrutiny, and these were counted for a four-day period both pre- and post-intervention. Items were also counted at a follow-up stage, but the intervening time varied across stores.

***Evaluating the Effects of EAS on Product Sales and Loss: Results of a Large-Scale Field Experiment*** – Hayes and Blackwood (2006)

Hayes and Blackwood studied the effect of concealed source-tagged EAS on personal grooming products in a retailer across several regions of the U.S. A purposeful sample of twenty-one stores with typical shrinkage rates for the company served as the treatment group. Two treatment conditions were tested - the proportion of goods tagged (50 per cent or 100 per cent) and whether there were tag detection gates installed. The outcomes investigated included item losses, shelf availability and store sales and data were collected using multiple methods, for example; weekly item counts; weekly sales; store shipments and store-level adjustments. Over 2,600 hours of CCTV video data were also used to observe the staff and customer response to EAS alarm activation.

***Trial of radio-frequency tags on CDs*–** Retailer A (2015)

This eight-week trial to introduce soft radio-frequency tags was conducted at a national, large U.K. retailer, who had identified that shrinkage for CDs equated to 0.91 per cent of sales. Existing safer-casing CDs (with a large transparent box around the product) were deemed unattractive and time-consuming for staff to remove at the point-of-sale. 20 stores were identified to receive the new ‘soft’ tags; 60 stores with similar average yearly sales, shrinkage and product ranges were selected as the control stores. A questionnaire was sent to the test stores – of which 14 responded – to collect feedback on the implementation of the new tags.

***Trial of radio-frequency tags on meat products* –** Retailer B (2015)

This trial was conducted at a national, large U.K. retailer, who had identified that shrinkage for meat, fish and poultry products equated to 1.22 per cent of sales. Radio-frequency (RF) source tags were introduced (in a phased manner) in 2015 for 62 specific red meat product lines. A group of similar, but untagged, products acted as the control. Economic data on the costs of tags and the staff time to apply the tags were presented, and the savings to the business were calculated based on projected shrinkage rates.

|  |  |  | | |  | | | | |  | |  | |
| --- | --- | --- | --- | --- | --- | --- | --- | --- | --- | --- | --- | --- | --- |
|  |  | **Mechanism** | | | **Moderator** | | | | | **Implementation** | | **Economics** | |
| Study author | Year | Risk | Reward | Effort | Staff/shop | Offender type | Tagging strategy | Item  type | Police & CJS | Staff | Tagging Strategy | Cost of tagging | Economic returns |
| Addis et al | 1993a | X |  |  |  |  |  |  |  |  | X |  |  |
| Addis et al | 1993b |  |  |  |  |  |  |  |  |  |  |  |  |
| Anon | 1982 |  |  |  |  |  | X |  |  |  | X | X |  |
| Bamfield | 1997 | X |  |  | X |  |  |  |  | X | X | X |  |
| Bamfield | 2012 |  |  |  | X | X |  |  |  |  |  |  |  |
| Bamfield | 1992 | X | X |  | X |  | X |  |  | X | X | X |  |
| Barua et al. | 2008 |  |  |  |  |  | X |  |  |  |  | X |  |
| Baumer & Rosenbaum | 1984 | X |  |  |  |  |  |  |  | X |  | X |  |
| Beck & Palmer | 2010 | X |  |  |  |  |  |  |  | X | X | X |  |
| Beck | 2002 | X |  |  | X | X |  |  |  | X | X | X |  |
| Beck | 2007 |  |  |  |  |  |  |  |  | X |  |  |  |
| Beck | 2008 | X | X | X |  |  |  |  |  |  |  | X | X |
| Beck | 2006 | X |  |  |  |  |  |  |  | X | X | X |  |
| Bender | 1997 | X |  |  | X |  | X | X |  | X | X | X | X |
| Bickman et al. | 1980 | X |  |  |  |  |  |  |  | X | X | X |  |
| Blackwood, R. and Hayes, R. | 2003 | X | X |  | X |  | X |  |  | X | X |  | X |
| Blackwood, R. and Hayes, R. | 2006 |  |  |  | X |  |  |  |  | X |  |  |  |
| Capers, C. | 2008 |  |  |  |  | X |  |  |  |  | X |  |  |
| Carmel-Gilfilen, C. | 2013 | X |  |  |  | X |  |  |  |  |  |  |  |
| ChainLink | 2014 |  |  |  |  |  |  |  |  |  | X | X | X |
| Conrad, A. | 1996 |  |  |  |  |  |  |  |  |  | X | X |  |
| Dawson, S. | 1993 |  |  |  |  |  |  |  |  | X |  | X |  |
| Dilonardo, R. | 1996 | X |  |  |  |  |  |  |  |  |  | X | X |
| Dilonardo & Clarke | 1996 |  | X |  |  |  |  |  |  |  | X | X |  |
| DiLonardo, R. | 2008 | X | X |  |  |  |  |  |  |  | X |  |  |
| Downs, D., Hayes, R. and Tallman, C. | 2011 | X |  |  | X |  |  |  |  |  |  |  | X |
| Farrington et al | 1993 | X |  |  |  |  |  |  |  | X | X | X |  |
| Giblin, M.B., Grottini, K. and Hayes, R. | 2015 | X |  |  |  | X | X |  |  |  |  |  |  |
| Gill, Bilby & Turbin | 1999 | X | X | X |  |  | X |  |  | X |  |  |  |
| Gohil &Trikha | 2013 |  |  |  |  |  |  |  |  |  |  | X |  |
| Gorshe et al | 2012 |  |  |  |  |  |  |  |  |  |  |  |  |
| Handford, M. | 1994 | X |  |  |  |  |  |  |  | X |  | X |  |
| Hayes & Blackwood | 2006 | X |  |  | X | X |  |  |  | X |  |  |  |
| Hayes | 1999 |  |  |  |  | X |  |  |  |  |  |  |  |
| Hayes | Unknown | X |  |  | X |  |  | X |  |  | X | X |  |
| Hayes & Cordone | 2006 |  | X |  | X |  |  |  |  |  |  |  |  |
| Huber | 2006 |  |  |  | X | X |  |  |  | X | X | X |  |
| Jones et al | 2005 |  |  |  | X |  |  |  |  | X | X | X |  |
| Klein | 1982 |  |  |  |  |  |  |  |  | X |  | X |  |
| Lindblom & Kajalo | 2011 | X |  |  |  |  |  |  |  | X |  |  |  |
| Loebbecke & Palmer | 2006 |  |  |  |  |  |  |  |  |  |  | X |  |
| Longmore-Etheridge | 1998 | X |  |  |  |  | X |  |  |  | X | X |  |
| Lottes | 1992 | X |  |  |  |  | X |  |  |  | X | X |  |
| Masuda | 1997 | X |  |  |  |  |  | X |  | X |  | X | X |
| Patton, | 2008 |  |  |  |  |  | X |  |  |  |  |  |  |
| Sensormatic | Unknown |  |  |  |  |  | X |  |  |  | X |  |  |
| Retailer A | 2015 |  |  |  |  |  |  |  |  | X |  | X |  |
| Retailer B | 2015 |  |  |  |  |  | X |  |  |  | X | X |  |
| Retailer C | 2015 |  |  |  |  |  |  |  |  |  |  | X | X |
| Retailer D | 2015 |  |  |  |  |  |  |  |  |  |  | X |  |

## Appendix 11: Summary of information relevant to Mechanisms, Moderators, Implementation and Economics reported in eligible studies
